# Non-Destructive Estimation of Total Chlorophyll Content of Apple Fruit Based on Color Feature, Spectral Data and the Most Effective Wavelengths Using Hybrid Artificial Neural Network—Imperialist Competitive Algorithm

**DOI:** 10.3390/plants9111547

**Published:** 2020-11-12

**Authors:** Razieh Pourdarbani, Sajad Sabzi, Mario Hernández-Hernández, José Luis Hernández-Hernández, Iván Gallardo-Bernal, Israel Herrera-Miranda

**Affiliations:** 1 Department of Biosystems Engineering, College of Agriculture, University of Mohaghegh Ardabili, Ardabil 56199-11367, Iran; s.sabzi@uma.ac.ir; 2 Faculty of Engineering, Autonomous University of Guerrero, Chilpancingo, Guerrero 39087, Mexico; joseluis.hernandez@itchilpancingo.edu.mx; 3 National Technology of México/Campus Chilpancingo, Chilpancingo, Guerrero 39070, Mexico; 4 Higher School of Government and Public Management, Autonomous University of Guerrero, Chilpancingo, Guerrero 39087, Mexico; Igallardo@uagro.mx; 5 Government and Public Management Faculty, Autonomous University of Guerrero, Chilpancingo, Guerrero 39087, Mexico; israelhm@uagrovirtual.mx

**Keywords:** non-destructive estimation, apples, spectroscopy, ANN, ICA algorithm, PSO algorithm

## Abstract

Non-destructive assessment of the physicochemical properties of food products, especially fruits, makes it possible to examine the internal quality without any damage. This is applicable at different stages of fruit growth, harvesting stage, and storage as well as at the market stage. In this regard, the present study aimed to estimate the total chlorophyll content using three types of data: color data, spectral data, and spectral data related to the most effective wavelengths. The most important steps of the proposed algorithms include extracting spectral and color data from each sample of Fuji cultivar apple, selecting the most effective wavelengths at the range of 660–720 nm using hybrid artificial neural network–particle swarm optimization (ANN-PSO), non-destructive assessment of the chemical property of total chlorophyll content based on color data, and spectral data using hybrid artificial neural network-Imperialist competitive algorithm (ANN-ICA). In order to assess the reliability of the hybrid ANN-ICA, 1000 iterations were performed after selecting the optimal structure of the artificial neural network. According to the results, in the best training mode and using spectral data and the most effective wavelength, total chlorophyll content was predicted with the R2 and RMSE of 0.991 and 0.0035, 0.997 and 0.001, 0.997 and 0.0006, respectively.

## 1. Introduction

Non-destructive prediction of physicochemical properties of fruits such as titratable acidity (TA), total soluble solid (TSS), texture, and chlorophyll will bring about dramatic changes in the food industry because of its speed, non-destructiveness, and availability online. Some of the internal properties that are of interest to consumers include soluble solid content (SSC), titratable acidity (TA), SSC/TA ratio, and texture. There are destructive and non-destructive methods to measure chemical properties. Destructive methods are traditional, offline, and time consuming (e.g., a pH meter to measure the acidity and a refractometer to measure the TSS (Pourdarbani et al. [1]; Flores et al. [2]; Pourdarbani et al. [3]; Pinheiro et al. [4]; Tigist et al. [5]; Huang et al. [6])). In recent years, researchers have tendeed to use online and non-destructive methods in the food industry (Pourdarbani et al. [7]; Mesa et al. [8]; Sirisomboon et al. [9]; Arendse et al. [10]). Some non-destructive methods include infrared spectroscopy (Magwaza and Opara [11]; Marcone [12]; Huang et al. [6]; Pourdarbani and Rezaei [13]); x-ray (Brecht et al. [14]); nuclear magnetic resonance imaging (Zhou et al. [15]); and Visible-Near Infrared (Vis-NIR) spectroscopy (Cavaco et al. [16]; Jamshidi [17]). Some researchers have studied the changes in the physical and chemical properties of different fruits during ripening (Arendse et al. [18]; Cavaco et al. [16]; Rungpichayapichet et al. [19]; Santagapita et al. [20]). Sabzi et al. [21]) obtained aerial images of Red Delicious apple to predict their maturity among four categories: immature, semi-ripe, ripe, and over-ripe. The proposed method was based on color properties and ANN optimized by genetic algorithms (GA). According to the results, the values of the receiver operating characteristic (ROC) and accurate classification rate were above 0.99 and 97.88%, respectively, for all classes. Fernandez-Novales et al. [22]) used a visible near infrared (VIS-NIR) spectrometer to estimate the amino acid content of grapes during ripening. Partial least squares (PLS) were used to create calibration, validation, and prediction models. The best performance (coefficient of determination (R2) ~ 0.60) was observed for asparagine (standard error of performance (SEP): 0.45 mg/L), tyrosine (SEP: 0.33 mg/L), and proline (SEP: 17.5 mg/L) in the range of 570–1000 nm and were observed for lysine (SEP: 0.44 mg/L), tyrosine (SEP: 0.26 mg/L), and proline (SEP: 15.54 mg/L) in the range of 1100–200 nm.

Information on the total content of fruit pigments is an important factor in evaluating and estimating the quality of agricultural products. Due to their strong antioxidant role, they are one of the factors that affect consumer choice. Lechaudel et al. [23]) non-destructively evaluated the maturity stages of fruits using the chlorophyll fluorescence method. Based on the degree-days method, physic-chemical measurements were performed. As fruits at the top of the canopy were more mature than fruits within the canopy, flesh color of shaded fruits were significantly greener. Moreover, parameters of chlorophyll fluorescence were significantly lower for fruits at the top of the canopy than those within the canopy. There were relationships between chlorophyll fluorescence parameters and maturity, predicted by CO_2_ on fruit still attached to trees that was independent of growth conditions such as the position of the fruit in the canopy. Steele et al. [24] predicted the leaf chlorophyll of grape. There was relationship between the red-edge chlorophyll index and chlorophyll content in the range of 710–720 nm (red-edge) and 755–765 nm (NIR). Merzlyak et al. [25] studied apple reflectance spectra (chlorophylls a and b, carotenoids, and anthocyanins) in anthocyanin-free fruit, where a close relationship was observed between reflectance at 550–700 nm with R2 of 0.95. In fruits with chlorophyll more than 5 nmoles/cm^2^, the reflection of 678 nm was not sensitive to the chlorophyll changes, while the reflection in the 550–650 nm and 690–705 nm was sensitive to changes in chlorophyll content. The reflectance in the range of 520–530 nm was mostly dependent on the carotenoid absorption. Solovchenko et al. [26] studied the changes in the total chlorophyll of apple. Samples were collected within the canopy during several seasons. Both cases of on-tree and off-tree ripeness were evaluated. Multi-season observations represented that on-tree patterns of both pigments as well as the rate of their ratio changes were closely related with off-tree chlorophyll content at harvest. Chlorophyll content was introduced as an appropriate index of fruit ripeness, but the changes in the content of both chlorophylls and carotenoids should be used to follow the ripening process in apple fruit on and off the tree, rather than the changes of each of the pigments alone. 

Based on the research background, it was found that various researchers worldwide are trying to provide different algorithms for the non-destructive estimation of the physicochemical properties of fruits. Thus, the aim of this study was to present three non-destructive algorithms for predicting the total chlorophyll content of Fuji apple as a criterion for determining the harvesting time using hybrid ANN-ICA. The first algorithm was based on color data extracted from various apple samples. The second algorithm was based on spectral data in the range of 660–720 nm. Finally, the third algorithm was based on spectral data related to the most effective wavelengths selected by the hybrid ANN-PSO.

## 2. Results

### 2.1. Physical, Chemical, and Color Features of Different Apple Samples

The physical, chemical, and color features of the apple samples using the criteria of maximum, minimum, mean, and standard deviation are given in Table 1.

### 2.2. Analysis of the Extracted Spectra of Samples

**Figure 1:**
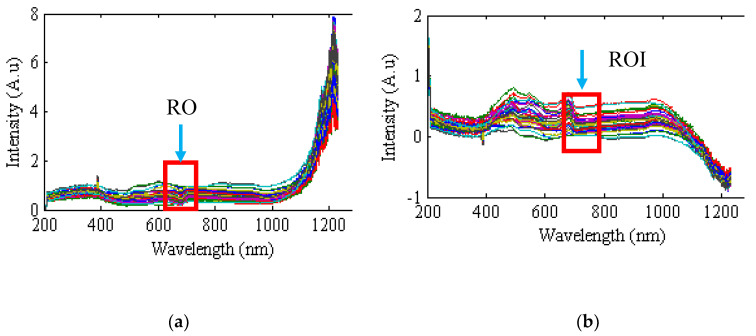
Spectral graphs of different samples in the range of 200–1300 nm. (**a**) Reflection spectrum. (**b**) Absorption spectrum.

Figure 1 represents the graphs for the reflection and absorption spectra of different apple samples in the range of 200–1300 nm. First, the reflection spectrum of each sample was extracted, and then, in order to establish a linear relationship with the molecular concentration of the samples, the reflection spectra were converted into the absorption spectra (see Equation (1)).

Absorption spectra = log(1/Reflectance spectra)(1)

As seen in Figure 1, there were different peaks, each of which had specific information about the internal features of the apple. The spectral range used in this paper is shown using a color box.

### 2.3. Non-Destructive Calculation of Total Chlorophyll Content Based on Spectroscopy

Using different criteria, the performance of ANN-ICA in the non-destructive estimation of total chlorophyll content was assessed based on spectral data of 660 to 720 nm. Table 2 gives the optimal structure of the hidden layers of the hybrid ANN-ICA.

Table 3 uses five different criteria including coefficient of determination (R2), sum squares error (SSE), mean absolute error (MAE), mean square error (MSE), and root mean squares error (RMSE) to evaluate the performance of the hybrid ANN-ICA in estimating the total chlorophyll content in 1000 iterations and also in the best training state at 660 to 720 nm. As can be seen, the mean value of the coefficient of determination in 1000 iterations was above 0.94 in the best training state of 0.9977. Additionally, different criteria related to hybrid ANN-ICA had small values. Therefore, it can be concluded that the proposed algorithm has the ability to predict total chlorophyll content using spectral data.

**Figure 2:**
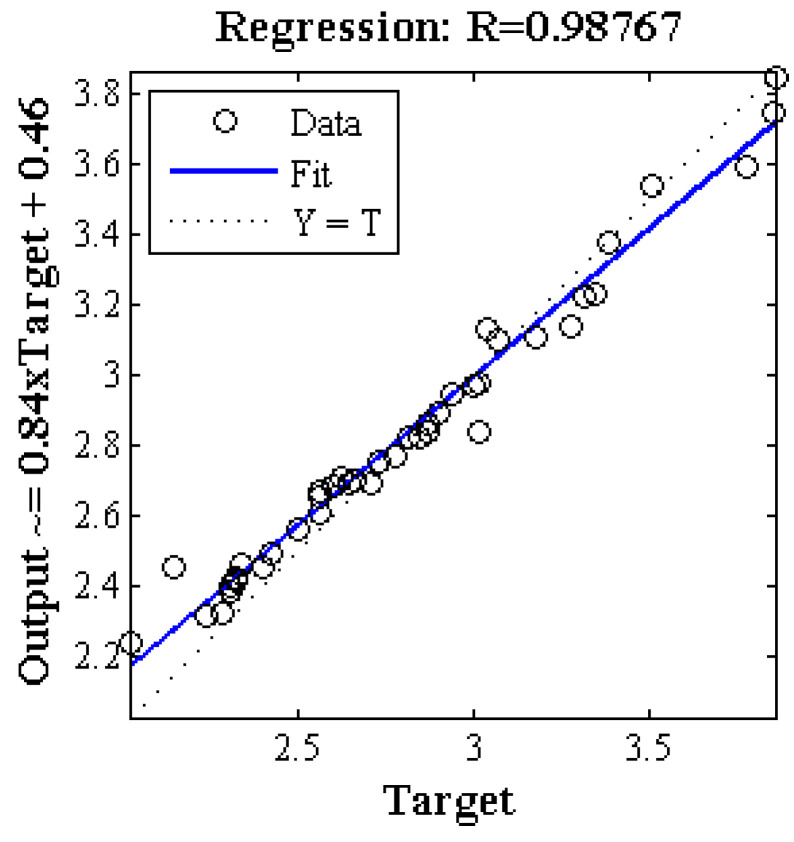
Analysis of the regression of the scatter plot between the mean estimated and the actual total chlorophyll content based on spectral data at the ranges of 660 to 720 nm in 1000 repetitions.

**Figure 3:**
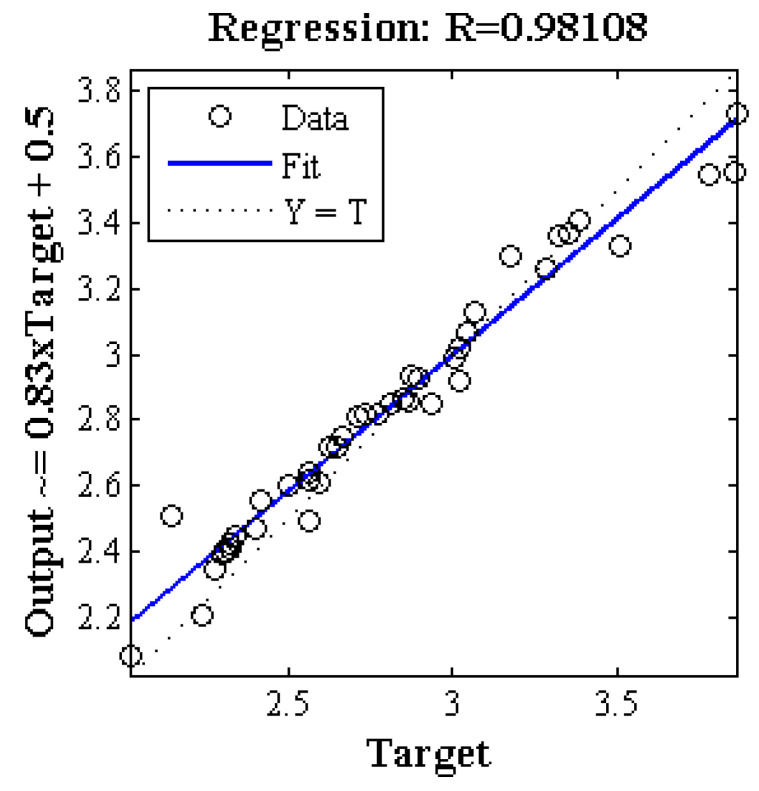
The regression of the scatter plot between the mean estimated and the actual value using the color features of a* b*.

Figure 2 represents the regression of the scatter plot between the mean estimated and the actual value (measured). As mentioned, 30% of the samples (14 samples) were randomly used for testing in each iteration. Therefore, in 1000 iterations, there weree 14,000 samples, and since the total number of apple samples was 45, there were 3111 = 14,000/45,000 iterations for each sample, and the mean value of the total chlorophyll content of each sample was calculated based on these iterations. As shown in Figure 3, the value of the regression coefficient (R) in this case was above 0.987, which indicates the high performance of the proposed method.

### 2.4. Non-Destructive Calculation of Total Chlorophyll Content Based on Color Features

Table 4 gives the optimal structure of the ANN used for non-destructive estimation of the total chlorophyll content using the color data of the first channel of L*a*b* color space (a*) and the second channel of L*a*b* color space (b*). As can be seen, in the optimal state, the artificial neural network has two hidden layers with the number of neurons of 13 and 17, respectively. Other specifications of the artificial neural network are shown in the table.

Table 5 used five different criteria to evaluate the performance of the hybrid ANN-ICA in estimating the total chlorophyll content at 1000 iterations as well as the best training state using the color features of a* and b*. As could obviously be seen, in the best training state, R2 was higher than 0.991 and the MSE was close to 0, which indicates the high performance of the hybrid ANN-ICA in estimating the chemical properties of chlorophyll.

Figure 3 demonstrates the regression of the scatter plot between the mean estimated and the actual value (measured). This figure implies that the value of the regression coefficient of ANN-ICA was above 0.981, indicating the high performance of the proposed method.

## 3. Discussion

### 3.1. Comparison of the Results of Algorithms Based on Color and Spectral Data for Non-Destructive Estimation of Total Chlorophyll Content

**Figure 4:**
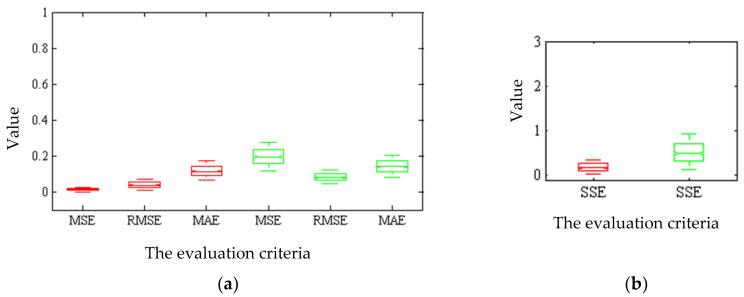
Box diagram of criteria assessing the hybrid ANN-ICA algorithm for non-destructive estimation of total chlorophyll content in 1000 iterations. The red boxes are related to the spectral data of 660 to 720 nm, and the green ones are related to the color features of a* and b* as input of the hybrid ANN-ICA algorithm. Four different criteria including mean square error (MSE) and root mean squares error (RMSE), mean absolute error (MAE), and sum squares error (SSE).

**Figure 5:**
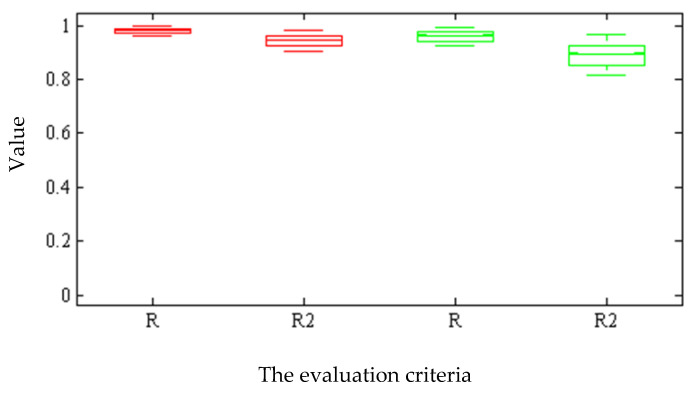
Box diagram of the coefficient of regression and determination of the hybrid ANN-ICA algorithm for non-destructive estimation of total chlorophyll content in 1000 iterations. The red boxes are related to the spectral data of 660 to 720 nm, and the green ones are related to the color features of a* and b* as input of the hybrid ANN-ICA algorithm. Two different criteria were used including the regression coefficient (R) and coefficient of determination (R2).

Figure 4 gives the box diagram of the criteria assessing the hybrid ANN-ICA algorithm for the non-destructive estimation of the total chlorophyll content using spectral and color data in 1000 iterations. The more compact the box diagram, the higher the reliability of the estimation because the compact diagram implies the closeness of the results in different iterations. When an ANN uses spectral data as input, box diagrams are more compact and have smaller values than color data. Figure 5 gives the box diagram of the regression coefficient of regression and determining of hybrid ANN-ICA algorithm in non-destructive estimation of chlorophyll. A comparison of Figure 4 and Figure 5 implies the superiority of the hybrid ANN-ICA algorithm in the case of spectral data used as input.

### 3.2. Selection of the Most Effective Wavelengths

If there are fewer wavelengths, the spectroscopy system is cheaper and more economical. This is why selecting the most effective wavelengths are so important. Table 6 shows the mean and standard deviation of the performance of the hybrid ANN-ICA algorithm based on the most effective wavelengths per 1000 iterations. As it is understood, for a case of nine effective wavelengths as the input of the neural network, the coefficient of determination was higher and the values related to the estimation error were less than in other cases.

**Figure 6:**
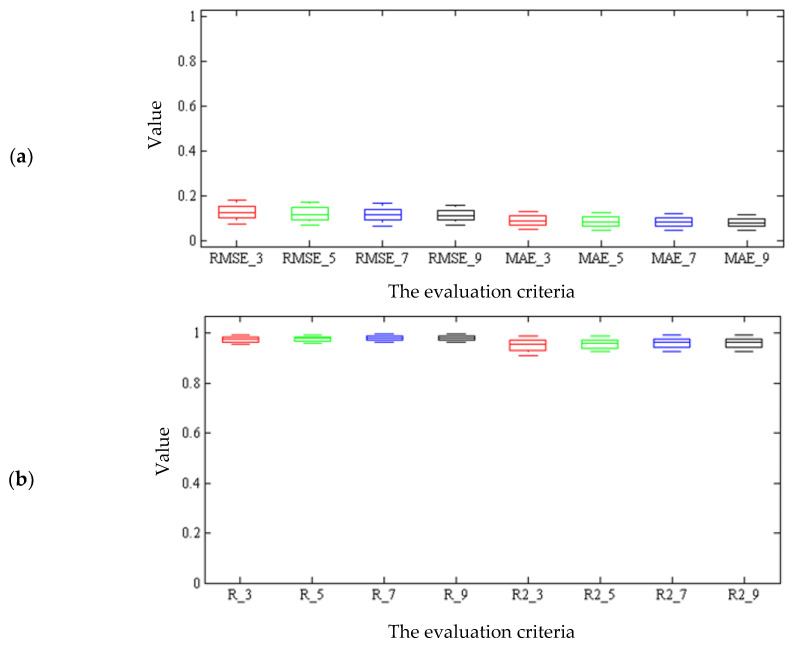
Box diagram of: (**a**) Root mean square error (RMSE) and mean absolute error (MAE) and (**b**) regression coefficient (R) and coefficient of determination (R2) of the neural network hybrid in the non-destructive estimation of chlorophyll. Subtitle numbers indicate the number of effective wavelengths.

Figure 6 represents the box diagram of the criteria assessing the performance of thee system and coefficients of determination of the hybrid ANN-ICA algorithm, respectively. As can be seen, the box diagrams of nine spectral data as the input of the hybrid ANN-ICA algorithm were more compact than others, indicating the high performance of the proposed method for non-destructive estimation of the total chlorophyll content.

### 3.3. Comparison of the Results Obtained in this Study with the Results of Other Researchers

In this section, the results of the proposed methods were compared with the results of other researchers for the non-destructive estimation of chlorophyll using the criterion of R2. As can be seen in Table 7, the value of the R2 of the proposed methods was higher than the other methods used by researchers. In general, the results of this study showed that by using both color and spectral data, it is possible to estimate the chlorophyll more accurately.

## 4. Materials and Methods

### 4.1. Data Collection

In order to train the proposed algorithm, the non-destructive estimator of the total chlorophyll content of Fuji apple should be tested against different samples. For this reason, 45 Fuji apple samples were collected from Kermanshah orchards at three stages of apple fruit growth. In fact, the approximate harvesting time was initially determined by various gardeners based on their experience. Then, 15 samples were collected 20 days before the given time, 15 samples were collected 10 days before the given time, and 15 samples were collected at the time of the harvest. The collected samples were immediately transferred to the laboratory to extract the color and spectral features.

### 4.2. Spectroscopy Configuration

**Figure 7:**
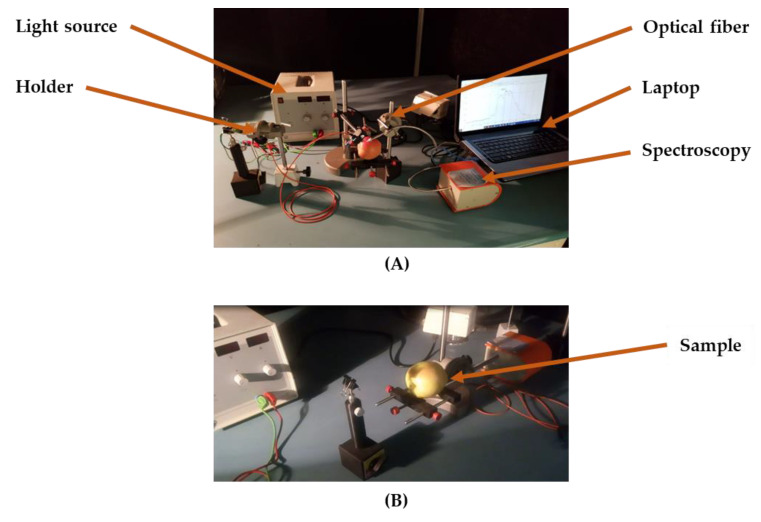
Setup of the spectroscopy system used in this study. (**A**) Spectroscopy components, (**B**) Sample for testing.

After the collection of different apple samples, spectral data of each sample were extracted using the spectroscopy configuration presented in Figure 7.

This hardware system had four main components including the laptop (Intel Core i3 CFI, 330M at 2.13 GHz, 4 GB of RAM), spectrometer (StrllarNet, 200 to 1100 nm and a resolution of 1 to 3 nm), light source SLI-CAL (StellarNet, USA) model of halogen, and two optical fibers for light transmission (Nicolai et al. [30]). Five random points on each apple were used for the analysis, and the average of their spectral data was considered in this research. The exact location of the optical fiber is not important because the radiation penetrates the apples.

### 4.3. Pre-Processing of Spectral Data Extracted from Samples

Due to the unwanted information related to the background and ambient light, the spherical shape of the samples and the different sizes of the samples, the spectral data included unwanted information. Therefore, pre-processing was required in order to achieve stable and reliable calibration models. In this study, first, the reflectance spectra were converted to absorption spectra in order to establish a linear relationship with the molecular concentration of the samples (Equation (2)).

Absorption spectra = log(1/Reflectance spectra)(2)

Next, baseline corrections were performed using the standard normal variate (SNV) with wavelet transform. Finally, the smoothing operation was performed by the Savitzki–Golay algorithm. The preprocessing operation was performed using Parles software. Parles is a chemometric software used for multivariate modeling and prediction. This software has the ability to transfer and pre-process the spectra received from different samples by various algorithms [31].

### 4.4. Extraction of Color Feature

After extracting the spectral data from different apple samples, we used the CR-400 colorimetric device (Konika Minolta, Japan) to extract the color features related to the three channels of the color space L*a*b* (García-Mateos et al. [32]).

### 4.5. Extraction of Total Chlorophyll Content

As chlorophyll levels change during fruit growth, this feature can be used to estimate ripening time with high accuracy (Costa et al. [33]; Amoriello et al. [34]). In order to extract the total chlorophyll content, the method by Betemps [29] was used. Equation (3) shows how total chlorophyll content is calculated.

(3) $$Chl_{a+b}=22.12E_{652.0}+2.71E_{665.2}$$

where *E* is the rate of absorption of the sample at the wavelength of the subtitle *E*. For example, *E*_652.0_ is the absorption at a wavelength of 652 nm.

### 4.6. Non-destructive Estimation of Total Chlorophyll Content

In order to estimate the total chlorophyll content using artificial data and spectral data, the hybrid ANN-ICA algorithm was used. The multilayer perceptron neural network (MLPNN) has several adjustable parameters, the optimal setting of which guarantees the high performance of the ANN in estimating the total chlorophyll content. These adjustable parameters include the number of layers, the number of neurons, transfer function, the back-propagation network training function, and back-propagation weight/bias learning function. The task of the imperialist competitive algorithm (ICA) is to optimally adjust these parameters. ICA is an algorithm based on cultural, social, and political evolution in which all countries are looking for the optimal public point to solve the optimization problem (Atashpaz-Gargari and Lucas [35], Abbaspour-Gilandeh et al. [36]). After selecting the optimal structure of the artificial neural network, 1000 iterations were performed due to the reliability of the ANN. For each iteration, 60% of the input data were randomly used to train the network, 10% were randomly used for validation, and 30% of the data were used randomly to test the network.

#### 4.6.1. Spectral Data Used in This Study

Given that in spectral graphs, the peaks in the visible areas are attributed to chlorophyll absorption (Cayuela [37]; Martínez-Valdivieso et al. [38]), the wavelength of 660–720 nm was used for the non-destructive estimation of total chlorophyll content.

#### 4.6.2. Color Data Used in This Study

In order to estimate of the total chlorophyll content, two color features of a* and b* were used.

### 4.7. Selection of the Most Effective Wavelength at the Range of 660–720 nm for Non-Destructive Estimation of the Total Chlorophyll Content

To develop a portable device for the non-destructive estimation of total chlorophyll content, it is necessary to use spectral data as little as possible. The reason is to increase the speed of calculation and reduce the cost of the production of a portable device. Therefore, the hybrid ANN-PSO algorithm was used to select the most effective wavelengths. The particle swarm algorithm (PSO) is a meta-heuristic algorithm that mimics the collective motion of birds to optimize various problems. This algorithm was first proposed by Kennedy and Eberhart [38]. Each answer to the problem is considered as a particle. Every particle is constantly searching and moving. The motion of each particle depends on three factors including the current position of the particle, the best position that the particle has ever had, and the best position that the whole set of particles has ever had (Kennedy and Eberhart [39]). Table 8 shows the structure of the artificial neural network used to select the optimal wavelengths.

### 4.8. Criteria for Evaluating the Performance of the Estimation System of Total Chlorophyll Content

In order to evaluate the performance of the hybrid ANN-ICA algorithm for non-destructive estimation of the total chlorophyll content, the statistical method of linear regression was used. Additionally, the criteria of the coefficient of regression (R), coefficient of determination (R2), mean square error (MSE), root mean square error (RMSE), and mean absolute error (MAE) were used. The formulas to calculate these parameters are as follows (Equations (4)–(8)):

(4) $$\mathrm{MSE}=\frac{1}{n}\overset{n}{\underset{s=1}{\sum }}{\left(X_{s}-Y_{s}\right)}^{2}$$

(5) $$\mathrm{RMSE}=\sqrt{\frac{1}{n}\overset{n}{\underset{s=1}{\sum }}{\left(X_{s}-Y_{s}\right)}^{2}}$$

(6) $$\mathrm{MAE}=\frac{1}{n}\overset{n}{\underset{s=1}{\sum }}\left|X_{s}-Y_{s}\right|$$

(7) $$R^{2}=1-\left\{\frac{\sum_{s=1}^{n}{\left(X_{s}-Y_{s}\right)}^{2}}{\sum_{s=1}^{n}{\left(X_{s}-X_{m}\right)}^{2}}\right\}$$

(8) $$\mathrm{MAE}=\frac{1}{n}\overset{n}{\underset{s=1}{\sum }}\left|X_{s}-Y_{s}\right|$$

where *n* is the number of samples in the test set; *X_s_* is the measured value of the property for the sample s; *Y_s_* is the estimated value of the property for sample s; and *X_m_* is the mean of the measured values of the property. Some research works also reported the regression coefficient, R, which is simply computed as the square root of R2.

On the other hand, given a certain accuracy measure (MSE, RMSE, MAE, or R2), since the experiment was repeated many times, it is interesting to report not only the mean value obtained, but also the standard deviation (SD) of this measure. This value indicates the stability of the method in different executions, the ideal situation being an SD near 0. Let us suppose an accuracy measure *M*, which is repeated m times, producing values *M_j_* for *j* = 1, …, *m*. The SD of this measure is defined as Equation (9):

(9) $$\mathrm{SD}\left(M\right)=\sqrt{\frac{1}{m-1}\overset{m}{\underset{j=1}{\sum }}{\left(M_{j}-\bar{M}\right)}^{2}}$$

where $\bar{M}$ is the average of the values of *M_j_*.

## 5. Conclusions

In this paper, using three types of data, namely, color data, spectral data of 660 to 720 nm, and spectral data related to the most effective wavelengths selected by the ANN-PSO algorithm, the amount of total chlorophyll content was estimated non-destructively. The most important results are as follows:

- Using the color features related to the color space L*a*b* (i.e., channel a* and channel b*), the total chlorophyll content was estimated with R2 above 0.991. Therefore, this chemical property can be predicted using an ordinary camera.
- The hybrid ANN-ICA algorithm uses spectral data of 660–720 nm to predict the total chlorophyll content with a higher coefficient than that of color data. The value of R2 for the hybrid ANN-ICA was above 0.9977.
- According to the results, while the hybrid ANN-ICA that used the spectral data related to the most effective wavelength had an almost identical coefficient of determination rather than the one that used the spectral data of 660–720 nm.
- Considering that hybrid ANN-ICA algorithms estimate the total chlorophyll content using the spectral data of the most effective wavelengths, it is possible to develop a portable device estimating this feature in orchards, which would lead to better management during storage and post-harvest operations.
- It is recommended that this method is used to estimate the different physicochemical properties of other fruits.

## Figures and Tables

**Table 1:** Physical, chemical, and color features of the apple samples.

Feature	Max	Min	Mean	SD
Length (mm)	64.42	46.73	55.40	3.41
Diameter (mm)	71.10	50.95	63.13	3.28
Weight (gr)	165.22	64.11	114.72	16.33
First channel of L*a*b*	80.54	66.84	76.21	2.59
Second channel of L*a*b*	−4.47	−15.48	−12.05	2.75
Third channel of L*a*b*	47.59	31.79	42.63	2.95
Total chlorophyll content (µgr/mL)	9.72	0.43	4.42	1.57

**Table 2:** Optimal values of multilayer perceptron (MLP) neural network parameters for hidden layers set by the Imperialist competitive algorithm (ICA).

Description	Optimal Values
Number of layers	First layer: 8, Second layer: 12
Number of neurons	2
Transfer function	First layer: tansig, Second layer: tansig
Back-propagation network training function	trainlm
Back-propagation weight/bias learning function	learnhd

**Table 3:** Results of different criteria for evaluating performance of Artificial Neural Network-Imperialist Competitive Algorithm (ANN-ICA) in estimating the total chlorophyll content using spectral data of 660–720 nm.

Description	R2	SSE	MAE	MSE	RMSE
Mean ± SD (1000 iterations)	0.947 ± 0.053	0.269 ± 0.419	0.092 ± 0.048	0.021 ± 0.032	0.127 ± 0.066
The best training state	0.997	0.014	0.026	0.001	0.033

**Table 4:** Optimal values of the Multi Layer Perceptron (MLP) neural network parameters for hidden layers set by Imperialist Competitive Algorithm (ICA) algorithm for non-destructive estimation of total chlorophyll content using the color data of a* and b*.

Description	Optimal Values
Number of layers	First layer: 13, Second layer: 17
Number of neurons	2
Transfer function	First layer: tansig, Second layer: tansig
Back-propagation network training function	trainscg
Back-propagation weight/bias learning function	learngdm

**Table 5:** Different criteria for evaluating the performance of the hybrid ANN-ICA in estimating the total chlorophyll content using the color features of a* and b*.

Description	R2	SSE	MAE	MSE	RMSE
Mean ± SD (1000 iterations)	0891 ± 0.047	0.659 ± 1.25	0.0151 ± 0.064	0.051 ± 0.096	0.206 ± 0.091
The best training state	0.991	0.046	0.043	0.0035	0.059

**Table 6:** Mean, standard deviation of ANN based on the most effective wavelengths in 1000 iterations.

Number of Features	Criteria	R2	SSE	MAE	MSE	RMSE
3	Mean ± SD (1000 iterations)	0891 ± 0.047	0.329 ± 0.0721	0.096 ± 0.046	0.025 ± 0.055	0.138 ± 0.078
	The best training state	0.996	0.011	0.023	0.0008	0.0029
5	Mean ± SD (1000 iterations)	0.946 ± 0.071	0.282 ± 0.0452	0.092 ± 0.041	0.022 ± 0.035	0.131 ± 0.067
	The best training state	0.998	0.019	0.031	0.002	0.038
7	Mean ± SD (1000 iterations)	0.95 3 ±0.036	0.229 ± 0.0258	0.088 ± 0.037	0.018 ± 0.019	0.122 ± 0.051
	The best training state	0.996	0.009	0.023	0.0007	0.026
9	Mean ± SD (1000 iterations)	0.954 ± 0.053	0.228 ± 0.280	0.087 ± 0.038	0.017 ± 0.022	0.121 ± 0.053
	The best training state	0.997	0.008	0.022	0.0006	0.025

**Table 7:** Comparison of the performance of the proposed methods with other methods.

Methods	Fruit	R2
Proposed method using spectral features	Apple	0.997
Proposed method using color features	Apple	0.991
(Ncama et al. [27])	Grapefruit	0.943
(Adebayo et al. [28])	Banana	0.978
(Betemps et al. [29])	Apple	0.934

**Table 8:** Structure of the ANN used to select the most effective wavelengths.

Description	Values
Number of layers	17
Number of neurons	1
Transfer function	tribas
Back-propagation network training function	trainr
Back-propagation weight/bias learning function	learnis
